# Evaluation of the Interaction between the *Poincianella pyramidalis* (Tul.) LP Queiroz Extract and Antimicrobials Using Biological and Analytical Models

**DOI:** 10.1371/journal.pone.0155532

**Published:** 2016-05-18

**Authors:** Thiago P. Chaves, Felipe Hugo A. Fernandes, Cleildo P. Santana, Jocimar S. Santos, Francinalva D. Medeiros, Délcio C. Felismino, Vanda L. Santos, Raïssa Mayer R. Catão, Henrique Douglas M. Coutinho, Ana Cláudia D. Medeiros

**Affiliations:** 1 Laboratory of drug assay and development, Universidade Estadual da Paraíba, Campina Grande, Paraíba, Brazil; 2 Department of Natural Sciences, Universidade Federal do Piauí, Bom Jesus, Piauí, Brazil; 3 School of Pharmaceutical Sciences, Universidade Estadual Paulista, Araraquara, São Paulo, Brazil; 4 Laboratory of Pharmacology, Universidade Estadual da Paraíba, Campina Grande, Paraíba, Brazil; 5 Laboratory of Research in Microbiology, Universidade Estadual da Paraíba, Campina Grande, Paraíba, Brazil; 6 Laboratory of Microbiology and Molecular Biology, Universidade Regional do Cariri, Crato, CE, Brazil; National University of Ireland - Galway, IRELAND

## Abstract

*Poincianella pyramidalis* (Tul.) LP Queiroz (Fabaceae) is an endemic tree of northeastern Brazil, occurring mainly in the Caatinga. Its medicinal use is widespread and is an important therapeutic option against diarrhea, dysentery, and respiratory and urinary infections, among other diseases. In this study we determined the chemical marker and evaluated the interaction between *P*. *pyramidalis* extract and a commercial antimicrobial through the use of biological and analytical models. To obtain the extract, an ethanol-water mixture (50:50 v/v) was used as solvent. It was nebulized in a *spray dryer* using colloidal silicon dioxide as a drying adjuvant. The extract (ENPp) was subjected to HPLC analysis to verify the presence of certain secondary metabolites. The Minimum Inhibitory Concentration (MIC) of the extract against Gram-negative bacteria was determined by broth microdilution and the MIC of synthetic antimicrobial drugs in the presence and absence of the extract. The antioxidant activity of ENPp was evaluated by the DPPH method. The compatibility between the antimicrobial and the extract was evaluated by thermal analysis (TG/DTA). The acute toxicity of the extract was evaluated in vivo in rodents. The results indicate significant additive action of the extract on synthetic antibiotics, considerable antioxidant activity and absence of toxicity. This extract shows high potential for the development of formulations for antimicrobial therapy when used with a vegetable-active ingredient.

## Introduction

The emergence of antibiotics was one of the greatest advances in modern medicine. These substances play a key role in the successful treatment of infections that used to take patients’ lives, and they also help increase life expectancy. However, the widespread and indiscriminate use of antibiotics has contributed to the emergence of resistant pathogens, including multidrug-resistant strains [1,2]. This problem has been aggravated in recent decades and has recently been recognized as one of the greatest threats to human health [3,4].

A particular concern is the case of the multiresistant Gram-negative bacteria. These microorganisms, which are intrinsically resistant to different antibiotics, have an outer membrane of low permeability that restricts access of the antimicrobial agents to their targets inside the cell, and this concern, together with the resistance mechanisms of the acquired-like efflux pump, enzymatic degradation, and change in drug target site, protect the bacteria against the deleterious effects of these agents [5]. A major threat to the global level caused by these bacteria are nosocomial infections because the treatment of patients in critical condition in an intensive care unit (ICU) or the treatment of other immunosuppressed patients becomes more complex if the condition is associated with increased morbidity and mortality [6,7].

The problem of increased antimicrobial resistance becomes even more menacing when the delay in the discovery and development of new antibiotics is taken into account. The number of such drugs is still quite limited, which endangers the essential role played by antibiotics in current medical practices [8].

The aforementioned problems urgently require new therapeutic strategies. Of special importance is the search for new drugs derived from biological sources in which molecules, predominantly secondary metabolites, contribute to their development [9]. Another approach to improving the efficacy of existing antimicrobials and suppressing the emergence of multidrug-resistant strains involves the use of products that potentiate the activity of these substances [10–12]. These products can improve the effectiveness of the antibiotic in eliminating or delaying the emergence of antibiotic resistance [13].

Plant extracts are known to have antimicrobial properties and may play an important role in therapeutic treatments. For this reason, a growing number of studies in different countries have been conducted to demonstrate the effectiveness of these extracts [14–16]. Besides the direct antimicrobial activity, plant species have been tested as potential adjuvants by modifying the microbial resistance [17,18].

Combinations of antimicrobial drugs and natural products of vegetable origin, in which these products act as adjuvants, constitute a promising approach for the treatment of infections. The natural products would replace at least a part of the synthetic substances in the formulations and would eventually reduce the undesirable effects of these substances in the human body [19].

*Poincianella pyramidalis* (Tul.) L.P. Queiroz (Fabaceae) is an arboreal species with wide distribution in the Brazilian semiarid region. Until recently, this species was known as *Caesalpinia pyramidalis* Tul., But due to a taxonomic update, it came to be called *P*. *pyramidalis* [20]. Its parts, especially its bark, leaves, and flowers, are used in traditional medicine for the treatment of several diseases such as influenza, cough, diarrhea, dysentery, respiratory infections, urinary infections, and inflammation in general [21–27]. Among the biological activities of *P*. *pyramidalis* described in the literature, we can highlight the antibacterial [28,29], antifungal [30], antioxidant [31], gastroprotective [32], anti-inflammatory, antinociceptive [33] and antihelminthic [34] activities.

This work is aimed to investigate the interaction between *P*. *pyramidalis* extract and antimicrobial drugs through the use of biological and analytical models.

## Material and Methods

### Plant material

Bark of *P*. *pyramidalis* were collected on the farm “Farinha”, municipality of Pocinhos, PB, Brazil (7°07´54.53´´S e 36°07´14.51´´O), in January 2014. A voucher specimen (CSTR 5036) was deposited in the herbarium of the Center for Health and Rural Technology at Federal University of Campina Grande.

### Preparation of extract

The plant materials were dried in an air circulation oven at 40°C. Subsequently it was ground in a knife mill with a particle size of 10 mesh. The hydroalcoholic extract obtained by extraction was assisted by ultrasound at 40°C for 60 min, using ethanol-water mixture (50:50 v/v) as solvent. Out below has been subjected to spray drying in a Mini *Spray Dryer* Labmaq PS-1, with onset temperature 120°C, air flow of 40 L min^-1^, drying air flow rate 3 ml min^-1^. The nebulized extract (ENPp) was dried with adjuvant using colloidal silicon dioxide (Aerosil 200^®^) at 20% on dry weight basis.

### Chemical assays

#### Determination of total polyphenols

The total polyphenol content of plant extracts was measured by Folin-Ciocalteu method [35]. The extracts were dissolved in distilled water to obtain a final concentration 200 μg mL^-1^. From each solution, a 1 mL aliquot was added to 1 mL of 1 mol L^-1^ Folin-Ciocalteu reagent (Sigma-Aldrich). This mixture remained undisturbed for 2 min before the addition of 2 mL of 20% (w/v) Na_2_CO_3_ solution and left undisturbed for 10 min. Thereafter the reading was performed Spectrophotometer Shimadzu, at 757 nm. The calibration curve was obtained with a stock solution of gallic acid (Sigma-Aldrich) (1000 μg mL^-1^), from which dilutions were made at concentrations between 1 and 40 μg mL^-1^.

#### Determination of total flavonoids

The total flavonoids were determined by the AlCl_3_ method [35]. The extracts were diluted with methanol at 1000 μg mL^-1^. To the 5 ml of each test solution was added the same volume of 2% (w/v) AlCl_3_ solution in methanol. This mixture remained undisturbed for 10 min before the UV spectrophotometric reading at 415 nm wavelength. The total flavonoids were determined by the calibration curve using quercetin (Sigma-Aldrich) as standard at concentrations between 2 and 30 μg mL^-1^.

#### Determination of condensed tannins

The content of condensed tannins was verified through the method described by Makkar and Becker [36] wherein 0.25 ml of the sample was added to 1.5 mL vanillin (Sigma-Aldrich) dissolved in methanol (4% w/v) and subsequently in 0.75 mL of concentrated HCl (37%). After the HCl addition, the tube content was shaken in water bath at 30°C for 3–4 seconds before being read on a spectrophotometer at a 500 nm wavelength. Catechin (Sigma-Aldrich) was used as standard at concentrations between 10 and 100 μg mL^-1^.

#### Determination of saponins

The quantification of total saponins followed the method described by Makkar et al.[37]. First, 250 μL of an 8% vanillin solution in ethanol was added to a 250 μL extract solution in 80% methanol; then 2.5 mL of 72% sulfuric acid were added. The tubes were incubated at 60°C in a water bath for 10 minutes and then transferred to an ice bath to rest for 4 minutes. The absorbance reading at 544 nm was performed against a blank consisting of the vanillin solution, 80% methanol and sulfuric acid. The calibration curve was obtained from a disogenin (Sigma-Aldrich) solution at concentrations between 100 and 500 μg mL^-1^.

#### Determination of major chemical compound

We used a liquid chromatograph Ultra Efficiency (UPLC), Shimadzu, equipped with two pumps model LC-20AD, autosampler SIL-20-AHT, oven column CTO-20A, detector with variable wavelength UV/Vis, model SPD-20A, controller CBM-20A, automatic computerized integrator with software LC Solution^®^. The stationary phase was composed of a column Gemini—NX C18 (250 x 4.60 mm, 5 μm). The mobile phase consisted of an isocratic mixture of acetic acid 0.1%: metanol (90:10, v/v). Analyses were performed under controlled temperature (30°C), using a flow 1 mL min^-1^ and injection volume of 20μL. All samples were amended with 0.45μm syringe filters diameter.

### Antioxidant activity

The antioxidant activity of ENPp was originally assessed by the ability of the antioxidant substances present in the sample to capture the free radical DPPH (1,1-diphenyl-2-picrylhydrazyl). The tests were conducted using the method described by Dhar et al. [38], with adaptations. Initially, the DPPH solution was prepared at 0.200 mM in ethanol. 500 μL of this solution was added to 500 μL of diluted extract in ethanol in concentrations ranging between 50 and 3.125 μg mL^-1^. The mixture remained at rest in the dark at room temperature for 30 minutes before the absorbance was read in a spectrophotometer at UV 517 nm. Gallic acid and quercetin were used as standards. The ability to scavenge DPPH radicals was calculated by the following equation:

$$Ability(\%)=\frac{\left({ABS}_{control}-{ABS}_{sample}\right)}{{ABS}_{control}}x\;100$$ (1)

where, ABS_control_ is the absorbance of the DPPH radical + ethanol; ABS_sample_ It is the absorbance of the DPPH radical + extract or standard.

The inhibitory concentration (IC_50_) and effective concentration (EC_50_) were estimated as described by Kroyer [39] and Prakash et al. [40]. The IC_50_ was determined by plotting the DPPH elimination ability against the logarithm of the concentration of the sample, while the EC_50_ was calculated using the following equation:

$${EC}_{50}\frac{{IC}_{50}}{[DPPH]{\mu g.mL}^{-1}}$$ (2)

### Microbiological assays

Were used standard strains American Type Culture Collection (ATCC) and clinical isolates of *Escherichia coli*, *Pseudomonas aeruginosa* and *Klebsiella pneumoniae* whose phenotypic profile is described in Table 1. The strains were maintained on slants of tubes with Mueller-Hinton Agar, and, before testing, cultured at 37°C for 24 hours, plates with the same culture medium.

**Table 1:** Bacterial strains used and their phenotypic profile of antimicrobial resistance.

Strains	Resistance
*E*. *coli* ATCC 25922	-
*E*. *coli* 401	AMC, CFL; ATM; CFO; NIT; CPM; SFM; NOR
*E*. *coli* 613	CFO; ATM; CFL; CAZ; CPM; AMP; GEN; NOR; CLI; TET; SFM.
*E*. *coli* 523	CIP, CLO, NOR, SFM, TET e AMP
*E*. *coli* 534	AMP; CFO; NOR; CAZ; ATM; TET; CPM; GEN; CFL; CLI; SFM.
*P*. *aeruginosa* ATCC 27853	-
*P*. *aeruginosa* 106	TOB; CFL; ATM; AMI; CPM; CFO; AMC; CFT;
*P*. *aeruginosa* 117	CFO; AMC; CFL; CFT; CAZ; SFM; NOR; GEN; CIP; TET; CPM.
*P*. *aeruginosa* 208	TOB; AMI; SFM; AMP; GEN; NOR; CLI; TET.
*K*. *pneumoniae* ATCC 4352	-
*K*. *pneumoniae* 110	AMC; CFO; CFL; NIT; CAZ; ATM; CFT; TET; NOR; CLI; SFM; AMP; GEN.

AMC = Amoxicillin + Clavulanic acid; CFL = Cephalothin; ATM = Aztreonam; CFT = Cefoxitin; NIT = Nitrofurantoin; CPM = Cefepime; NOR = Norfloxacin; AMP = Ampicillin; CIP = Ciprofloxacin; CFO = Ceftriaxone; CAZ = Ceftazidime; GEN = Gentamicin; CLI = Clindamycin; TET = Tetracycline; CLO = Chloramphenicol; TOB = Tobramycin; AMI—Amikacin; SFM = Sulfamethoxazole + trimethoprim.

#### Determination of Minimum Inhibitory Concentration (MIC)

The minimum inhibitory concentration (MIC) was determined by a microdilution method in 96-well plates using Mueller-Hinton broth [41]. Colonies of microorganisms were suspended in a 0.9% saline solution, and by a spectrophotometric method at 625 nm, the suspension adjusted to a final concentration of 5 x 10^6^ CFU mL^-1^. Serial dilutions of the extract in the range of 1000 to 2.4 μg mL^-1^ and antibiotics in the range of 2500 to 2.4 μg mL^-1^ were performed. Dimethyl sulfoxide (DMSO) 10% was included as a negative control. The plates were incubated at 37 ± 1°C for 24 hours. Bacterial growth was indicated by addition of 20 μL of 0.01% aqueous resazurin solution (Sigma-Aldrich) with incubation at 37 ± 1°C for 2 h. MIC values were identified as the lowest concentration in which no bacterial growth is visible. The assays were performed in triplicate.

#### Modulation of antimicrobial resistance

Evaluation of extracts as modulators of antibiotic resistance was performed according to Coutinho et al.[42]. The MIC of the antibiotic was determined in presence and absence of sub-inhibitory concentrations (MIC/8) of EESb. Plates were incubated as described above and each assay was performed in triplicate.

### Evaluation of acute toxicity

The study was carried out in strict accordance with the Standard Operating Procedures (Laboratory of Pharmacology at the State University of Paraiba, Campina Grande, Brazil) approved by veterinarian, which monitored frequently the animals by physical condition assessments of the health. All efforts were exerted in order to reduce the suffering of experimental animals. The protocol was approved by the Ethics Committee on Animal Use of Faculty of Medical Sciences of Campina Grande (No: 5618092015). Disease-free albino Wistar rats (Rattus norvegicus) (6–8 weeks age and 200–220 g weight) were used for this study. The animal house were obtained of Federal University of Paraíba, João Pessoa, Paraíba, Brazil. The animals were housed in rat standard plastic cages (n = 6) with stainless steel coverlids and wood shavings. All rats underwent a period of at least 7 days of acclimatization prior to the procedure, being socialized with contact including humans. The animals were handling with care to minimize stress. The researchers confirm that the laboratory had a protocol in place for the use of humane endpoints in cases where animals became severely ill or moribund during the experiment, but no had death or behavioral changes in animals. They remained in polypropylene boxes, in single sex groups, at room temperature (22°C ± 3°C) and humidity (50% ± 20%) and 12 hrs light/dark cycles. The animals received standard laboratory pellets and water ad libitum both for the adaptation period (7 days) and during the trial, except the period of 12 hours prior to the experiment in which the access to food was restricted. Throughout the experiments, all of the animals received humane care according to the ‘‘Guide for the Care and Use of Laboratory Animals” prepared by the National Academy of Sciences [43].

The animals were divided in groups of 6 males and 6 females, which received, orally, the dose of extract to 2000 mg kg^-1^. A control group was treated with saline, the same used to resuspend the extract. Immediately after administration of the extract, the animals were evaluated behaviorally carefully during the first 4 hours, as recommended by the handshake protocol and evaluation of clinical signs of OECD [44] and daily for 14 days after administration. They were also observed the consumption of water and feed. Animals were sacrificed under anesthesia with ketamine/xylazine (0.5 mL of 100 mg mL^-1^ ketamine combine with 0.05 mL of 20 mg mL^-1^ xylazine) at a dosage of 0.55 mL/ 100g body weight (b.w.). After sacrifice was carried out weighing and macroscopic analysis of the viscera (liver, kidneys, spleen, lungs and heart).

### Statistical analysis

The results of the microbiological tests were expressed as a geometric mean. It was applied to a two-way analysis of the variance, followed by the Bonferroni post-test, and was applied to toxicity testing through an analysis of the variance with Tukey's post-test using GraphPad Prism 5.0 software.

### Thermal analysis

The thermoanalytical profiles were obtained using a simultaneous TG-DTA analyzer, model DTG-50 (Shimadzu). Samples (5.0 ± 0.2 mg) were accommodated in a platinum crucible, and subjected to a heating program from 30 to 900°C, at 10°C min^-1^, in inert nitrogen atmosphere (50 mL min^-1^). The samples consisted of antibiotics and extract analyzed separately and in binary mixtures, the proportion 1:1.

The DTA module is calibrated with indium standard (mp = 156.6°C). And the calibration of TG module, was used a standard calcium oxalate monohydrate. Curves were analyzed in the TA60 software, version 2.21.

## Results and Discussion

### Chemical assays

The secondary metabolite content is shown in Table 2. The content of the total number of polyphenols and tannins is high compared to the total flavonoids. Although the method above is a quantitative method, it fails to predict the composition of each individual compound as well as the possible quantification of nonphenolic compounds [45].

**Table 2:** Content of secondary metabolites present in *P*. *pyramidalis* extract obtained spectroscopy in the visible region.

Metabolites	Concentration
Total polyphenols	36.94 ± 0.45^1^
Total flavonoids	19.09 ± 0.78^2^
Condensed tannins	59.08 ± 0.69^3^
Total saponins	328.43 ± 1.95^4^

^1^Gallic acid equivalent (GAE)

^2^Quercetin equivalent (QE)

^3^Catechin equivalent (CE)

^4^Disogenin equivalent (DE).

Other studies carried out using the phytochemical with *P*. *pyramidalis* extracts revealed the presence of secondary metabolites such as saponins, ursolic acid, sitosterol, Cinnamic derivatives, flavonoids, quercetin, proanthocyanidins, catechin, gallic acid, and ellagic acid [29,46,47].

**Fig 1:**
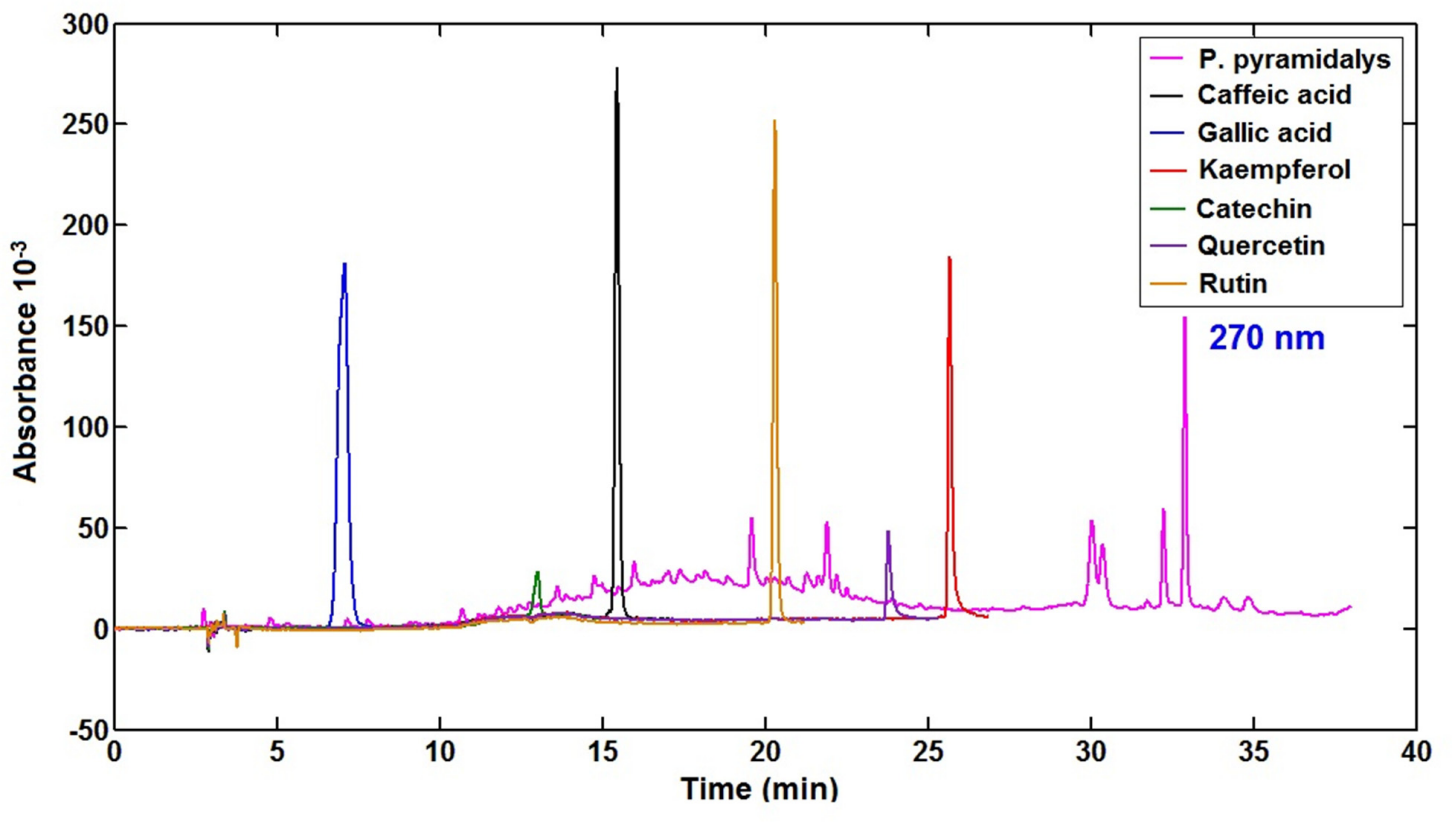
Chromatograms of nebulized extract of *P*. *pyramidalis* showing its chemical marker.

Identification of the major chemical compound using liquid chromatography was performed, and, based on the retention time parameter (TR), was compared with the TR values for the analyzed standards of gallic acid, catechin, quercetin, rutin, and kaempferol, with the RT values of peaks observed in the EEPp. The results indicated the presence of gallic acid, which can be used as a chemical marker of *P*. *pyramidais* (Fig 1). In a study conducted by Santana et al. [48] with the ethanol extract of the plant, the researchers found the presence of rutin, which was absent in this study. This difference may be related to parameters such as the place and time of collection and the stage of plant development, among other factors [35].

### Antioxidant activity

The search for substances that might reduce the risk of developing chronic diseases caused by oxidative stress [49] is increasing. This risk includes several pathological and toxicological processes such as aging, transformation, cell killing, cancer induction, autoimmune diseases, and heart diseases, among others [50,51]. Among the most promising substances in the study are those of vegetable origin, such as polyphenols, flavonoids, alkaloids, terpenoids, carotenoids, etc. [52–54].

The scavenging capacity of the free radicals of *P*. *pyramidalis* extract was compared through its ability to eliminate the DPPH radical. The data from these tests are shown in Table 3, expressed as a percentage of the inhibition of DPPH, IC_50,_ and EC_50_. When looking at the percentage of the inhibition of DPPH, we found that the extract showed a high inhibition rate, with no statistically significant difference between it and gallic acid. On the other hand, the IC_50_ and EC_50_ extract was significantly higher than the standards. Its inhibition rate may be related to the fact that the extract is a complex mixture of substances as the patterns are pure substances.

**Table 3:** Antioxidant activity of the extract and standards towards DPPH.

	DPPH (%)	IC_50_ (μg mL^-1^)	EC_50_ (U.A.)
Gallic acid (10 μg mL^-1^)	75.28^a^	5.99 ± 0.24^a^	0.0760 ± 0.0030^a^
Quercetin (10 μg mL^-1^)	36.84^b^	13.75 ± 0.24^b^	0.1743 ± 0.0030^b^
Extract (50 μg mL^-1^)	79.71^a^	28.11 ± 0.68^c^	0.3564 ±0.0086^c^

a, b, c—different letters in the same column mean statistically significant differences (p < 0, 01).

There are references in the literature that mention that *P*.*pyramidalis* has a good number of phenolic compounds [46]. These substances are very important components of plant extracts and contribute directly to the elimination of radicals because of their hydroxyl groups [55,56]. Gallic acid, found in *P*. *pyramidalis* extract, is one of these compounds, and there are reports in the literature of its antioxidant properties [57–59].

### Antimicrobial and modulatory activities

As for antimicrobial activity, the ENPp had no clinically significant effect on any of the studied strains. Dall'Agnol et al. [60] and Rios and Recio [61] reported that plant extracts are considered inactive with MIC > 1 000 μg mL^-1^; they are low-activity holders with CIM between 500 and 1000 μg mL^-1^ and show moderate activity with MIC values between 100 and 500 μg mL- ^1^ and good activity when MIC is ≤ 100 μg mL^-1^. The latter activity levels have good potential for the determination and purification of active compounds. The absence of antimicrobial activity presented here is consistent with the study conducted by Silva et al. [62], who detected the inactivity of the ethanol extract of *P*. *pyramidalis* on standard strains and clinical isolates of *E*. *coli*, *P*. *aeruginosa*, and *K*. *pneumoniae*.

Gallic acid, a chemical marker of ENPp, was also subjected to microbial susceptibility testing and showed no significant activity on strains tested in this study (MIC > 1000 μg mL^-1^). This result corroborates the study by Chanwitheesuk et al. [63], in which the gallic acid also presented MIC > 1000 μg mL^-1^ on several bacterial strains, Gram positive, Gram negative, and fungal. A similar result was observable in the study of Jayaraman et al. [64] in tests with standard and multidrug resistant strains of *P*. *aeruginosa*. Borges et al. [65], in trials with *E*. *coli*, *S*. *aureus*, and *Listeria monocytogenes*, obtained MIC values > 1000 μg mL^-1^. However, against *P*. *aeruginosa*, the gallic acid showed MIC = 500 μg mL^-1^. Other studies report MIC values for gallic acid that can be considered active. Sanchez-Maldonado et al. [66] obtained MIC = 490 μg mL^-1^ against *E*. *coli*. Vaquero et al. [67], using the agar diffusion method, obtained inhibition halos against standard strains of *E*. *coli* (ATCC 35218 and ATCC 25922), with gallic acid concentrations ranging between 200 and 1000 μg mL^-1^. The discrepancy between the findings of this and other studies may be related to the method used to determine the antimicrobial activity, as well as the phenotypic profile of the strains used.

The resistance presented by Gram-negative bacteria is possibly related to the presence of the outer membrane of the bacterial wall, which forms a semipermeable barrier composed mainly of phospholipids, lipopolysaccharides, and proteins. This barrier hinders the passage of antimicrobial drugs and is linked to the high intrinsic resistance of these bacteria [68,69]. Beyond this barrier, these microorganisms may have other mechanisms that prevent the antimicrobial from reaching the target. It is able to detect the change in the composition of the outer membrane, eliminating porins, induction efflux pumps, and enzymatic degradation of the antimicrobial [70,71].

**Fig 2:**
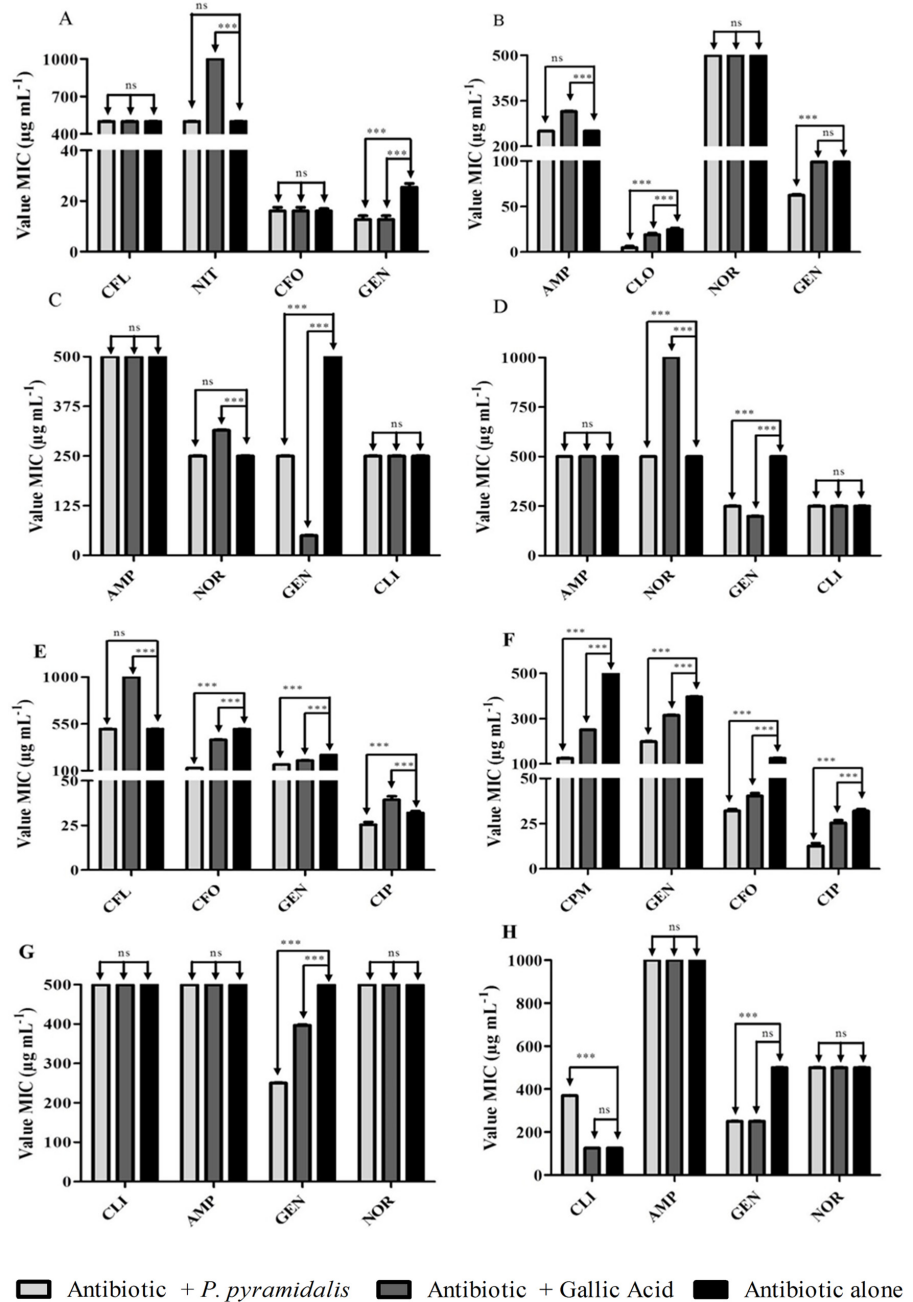
Modulatory activity of ENPp against of Gram negative strains. A—*E*. *coli* 401; B—*E*. *coli* 523, C—*E*. *coli* 613; D = *E*. *coli* 534; E—*P*. *aeruginosa* 106; F—*P*. *aeruginosa* 117; G—*P*. *aeruginosa* 208. H—*K*. *pneumoniae* 110. ***—statistically significant with P value < 0.001; ns–not statistically significant value with P > 0.05. CFL = Cephalothin, NIT = Nitrofurantoin, CF = Ceftriaxone, GEN = Gentamicin, CLI = Clindamycin, AMP = Ampicillin, CLO = Chloramphenicol, NOR = Norfloxacin, CIP = Ciprofloxacin, CPM = Cefepime.

In modulatory activity, assays have calculated a subinhibitory concentration (MIC / 8) of the extract, which are associat with antibiotics. Because the extract did not prevent efficacy, CIM considered for this calculation was the highest concentration tested and has, therefore, a subinhibitory concentration of 125 μg mL^-1^. In Fig 2A, 2B, 2C and 2D, we can see that, after combination with both the extract and gallic acid, there was a significant increase in the gentamicin activity on the *E*. *coli* strains 401, 534, and 613. Against *E*. *coli* 523, chloramphenicol reduced MIC in both associations, while gentamicin, only in association with the extract. Moreover, the association with gallic acid caused a significant increase in nitrofurantoin (*E*. *coli* 401), ampicillin (*E*. *coli* 523), and norfloxacin (*E*. *coli* 534 e 613). In assays with *P*. *aeruginosa* strains (Fig 2E, 2F e 2G), we found the additive effect of the extract and gallic acid in the action of gentamicin on all strains tested, ceftriaxone (*P*. *aeruginosa* 106 e 117), ciprofloxacin, and cefepime (*P*. *aeruginosa* 117). The extract also led to a significant reduction of ciprofloxacin, the MIC for *P*. *aeruginosa* 106, while the opposite effect resulted in gallic acid. Also, on this same strain, the association with gallic acid increased MIC of cephalothin. In assays with *K*. *pneumoniae* (Fig 2H), the antibiotic whose activity has intensified in both associations, was gentamicin. However, there was a significant increase in CIM clindamycin when it was added to the extract.

The best results in the interactions with the extract or gallic acid were observed in assays with gentamicin, whose CIM on all strains was reduced. This antimicrobial belongs to the class of aminoglycoside, which in turn is the class to which the most commonly used anti-infectious agents in clinical practice belong [72]. They are able to interact with different portions of rRNA, causing deleterious effects on mRNA translation process polypeptide causing inhibition of protein synthesis or production of defective proteins [72–74]. The main mechanisms of resistance to these antimicrobial agents include the enzymatic degradation (N-acetylation, adenylation, or O-phosphorylation), reduction in the intracellular concentration of the antibiotic by changes in membrane permeability and transport by active efflux; changes in the target 30S ribosomal subunit; and changes in the antimicrobial binding site [75,76].

A particularly important feature presented by the aminoglycosides is the ability to act synergistically with other drugs [76]. This feature is particularly important in the search for new therapeutic alternatives because of the emergence of resistant strains and toxicity caused by these drugs [77]. In the literature, there are studies that demonstrate the resulting synergism of the associations between β-lactams and aminoglycosides [78,79] and between aminoglycosides and natural products [80–82].

The combination of plant extracts and antibiotics is well documented in the literature. In many cases, this association led to an additive effect, resulting in improved antimicrobial activity against various multidrug-resistant strains [83–88].

The reduction in the MIC of gentamicin after association with ENPp, or its chemical marker, may be related to the promotion of drug entry into the bacterial cell. Gallic acid acts on cell membranes, leading to irreversible changes in its characteristics relating to intra- and extracellular permeability. The chemical-physical properties it is altered by changes in the hydrophobicity and decreased negative charge of the surface. It favors the occurrence of breaks or the formation of pores, with consequent loss of essential intracellular components for the bacterial life [65].

The ENPp as various types of plant extracts and phytochemicals presents a wide variety of compounds; therefore, other mechanisms may be related to the additive effects of the combination of antibiotics and ENPp. A wide variety of compounds present in the extract can act on different targets [89] and may be in one or multiple targets at once. Among the mechanisms by which the extracts can interfere with microbial growth is the inactivation of enzymes, transport and receptor proteins, and DNA/RNA, besides acting in the suppression of bacterial resistance mechanisms [90–92]. Moreover, the said plurality of compounds and their mechanisms of action provide a low risk for increasing resistance because they afford the greatest difficulties for microbial adaptation [93].

### Acute toxicity

During the 14 days following the statement of administration, there was no death among the animals, making it impossible to calculate the LD_50_. There was also no change in the behavior of the animals. When the consumption of water and feed and the average weight of the organs of animals were evaluated, there was no statistically significant difference between the group treated with the extract and the control group (P <0.05).

### Thermal analysis

#### Differential thermal analysis (DTA)

The DTA curve of ENPp shows three processes of the sample phase transitions. The first, endothermic at 53.36°C, is possibly related to loss of water, solvent (ethanol), or volatile compounds in the sample. The following two peaks are exothermic of crystallization: the second at 348.30°C and the third at 425.17°C (Table 4).

**Table 4:** DTA data of ENPp, antibiotics and their binary mixtures.

Samples	Peak 1			Peak 2			Peak 3			Peak 4			Peak 5		
	*T*_*peak*_/°C	∆*H/* J/g-^1^	*T*_onset and endset_/°C	*T*_*peak*_/°C	∆*H/* J/g-^1^	*T*_onset and endset_/°C	*T*_*peak*_/°C	∆*H/* J/g-^1^	*T*_onset and endset_/°C	*T*_*peak*_/°C	∆*H/* J/g-^1^	*T*_onset and endset_/°C	*T*_*peak*_/°C	∆*H/* J/g-^1^	*T*_onset and endset_/°C
ENPp	53.36	100.35	33.37–72.64	348.30	42.88	290.46–347.17	425.17	3.530.00	396.42–439.85	-	-	-	-	-	-
NOR	179.69	6.36	177.15–185.30	223.84	152.22	220.73–230.79	-	-	-	-	-	-	-	-	-
NOR + ENPp	179.36	1.88	176.94–182.26	219.51	36.92	210.08–224.10	428.63	4.42	423.22–434.30	612.19	601.79	576.34–680.96	-	-	-
AMP	63.52	12.15	59.68–68.27	218.30	67.97	213.45–227.17	363.24	125.99	354.77–373.46	-	-	-	-	-	-
AMP + ENPp	63.85	3.65	60.58–67.73	218.92	17.85	210.41–226.45	397.58	55.13	372.50–405.56	-	-	-	-	-	-
CIP	144.45	276.67	119.24–157.21	318.40	462.48	306.38–330.48	-	-	-	-	-	-	-	-	-
CIP + ENPp	134.90	124.53	112.28–147.43	296.64	47.36	293.81–310.86	405.53	10.57	390.24–411.35	430.63	10.50	418.69–440.96	-	-	-
NIT	272.07	244.79	268.61–276.39	307.84	8.73	277.92–315.87	-	-	-	-	-	-	-	-	-
NIT + ENPp	263.06	236.61	250.07–280.60	423.83	20.25	417.19–432.47	-	-	-	-	-	-	-	-	-
CFL	167.27	22.30	153.32–177.02	212.63	159.74	204.38–220.80	564.06	202.60	541.67–593.37	-	-	-	-	-	-
CFL + ENPp	81.02	255.21	36.14–170.68	211.66	42.64	204.49–219.80	-	-	-	-	-	-	-	-	-
CLI	51.85	121.54	34.88–67.37	76.84	30.37	71.36–87.41	207.50	28.64	206.85–215.42	241.92	41.42	218.79–251.80	-	-	-
CLI + ENPp	59.74	63.52	44.60–74.52	239.84	272.81	202.73–288.80	326.21	6.51	319.90–333.10	-	-	-	-	-	-
CFO	80.90	46.28	61.73–90.11	148.22	32.12	138.06–155.45	270.27	446.45	261.72–285.21	-	-	-	-	-	-
CFO + ENPp	85.12	24.52	76.96–94.34	154.40	20.64	146.02–161.92	273.23	553.49	255.18–300.31	356.14	180.95	350.89–417.65	546.50	533.03	533.42–584.77
GEN	75.05	350.04	42.44–112.06	252.84	250.41	242.03–278.10	299.71	194.99	284.83–331.94	-	-	-	-	-	-
GEN + ENPp	67.93	205.46	40.37–101.76	252.46	155.79	241.78–276.63	299.15	54.24	287.22–327.06	-	-	-	-	-	-
CPM	50.13	29.54	33.74–62.42	117.38	25.56	107.28–125.85	182.04	39.98	158.37–195.67	645.78	473.32	607.25–659.49	-	-	-
CPM + ENPp	69.05	112.11	37.33–86.13	108.11	8.26	98.95–116.19	194.00	4.55	192.92–203.86	-	-	-	-	-	-
CLO	184.87	225.63	164.22–217.94	279.09	133.40	258.62–301.24	576.71	328.00	522.61–617.78	-	-	-	-	-	-
CLO + ENPp	70.39	41.07	37.72–119.58	179.93	61.52	165.54–225.48	-	-	-	-	-	-	-	-	-

**Fig 3:**
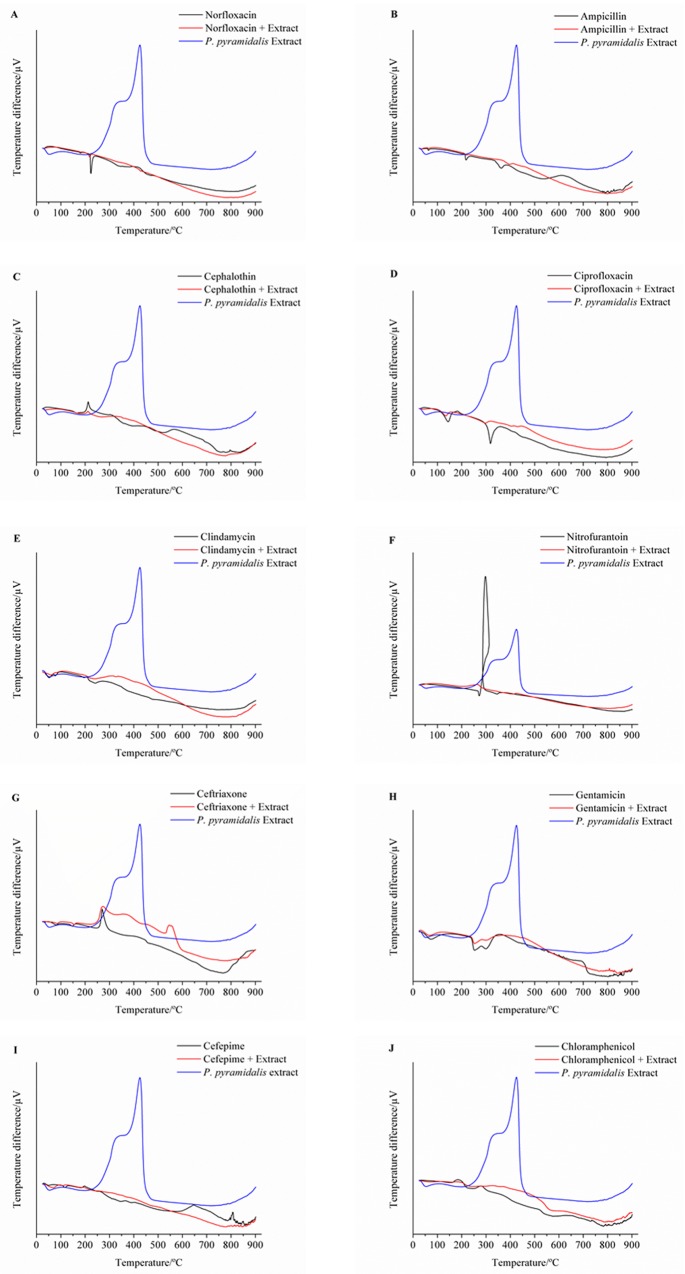
DTA curves of nebulized extract of *P*. *pyramidalis*, antimicrobials and their binary mixtures. (A) Norfloxacin, (B) Ampicillin, (C) cephalothin, (D) Ciprofloxacin (E) Clindamycin, (F) Nitrofurantoin, (G) Ceftriaxone, (H) Gentamicin, (I) Cefepime, (J) Chloramphenicol.

In the DTA curve of norfloxacin, the first peak of the endothermic nature at 179.69°C was observed. The second peak endotherm occurred at 223.84°C, which corresponds to the delay of the drug fusion process that occurs between 220.00 and 221.00°C [94,95]. In the binary mixture of antibiotic + extract, we observed that the first endothermic peak occurred at a temperature of 179.36°C, which is characteristic of the drug. The second peak was in 219.51°C, which corresponds to the anticipation of the melting process of the drug. From the third, occurring in 428.63°C, the thermal sample decomposition process begins. In the mixture, there was a change in the heat peaks in advance of the drug melting temperature and suppression of peaks observed in the extract. These changes are possibly due to interactions between the extract and antibiotic (Table 4/Fig 3).

Four events were recorded in the DTA curve of ampicillin. The first, which were endothermic, recorded in temperatures of 63.52°C, related to the dehydration sample; the second event was in 218.30°C, which probably corresponds to the delayed melting process, with accompanying decomposition, of one of its anhydrous forms [95,96]; the third, at 363.24°C, was the beginning of the decomposition of ampicillin. In the curve produced with the mixture, we observed that the first two endothermic peaks of the drug were maintained at the same temperature but with less reaction heat involved in the endothermic processes. The removal of the exothermic peaks of the drug and the extract in the binary mixture curve is an indication of a strong chemical interaction between the components of the sample (Table 4/Fig 3B).

The DTA curve of cephalothin showed an endothermic peak at 167.27°C, probably related to the sample fusion process between 160.0 and 160.5°C [95], and an exotherm at 212.63°C, which corresponds to their decomposition processes [97]. With the mixture produced from the extract, a glass transition was observed at a temperature of 151.33°C and an exothermic peak at 211.66°C, both associated with the degradation of the mixture (Table 4/Fig 3C).

In the DTA curve of ciprofloxacin, two events occur, the first, of an endothermic nature, was observed at 144.45°C, corresponding to the loss of the acetylene group (C_2_H_2_) of the drug [98]. The second, at 318.40°C, corresponds to the melting process of the drug, which occurs between 318.0 and 320.0°C [95,99]. In the curve of the mixture, four endothermic peaks were observed. The first, at 134.90°C, resulted from the sample moisture loss and the drug group C_2_H_2_[98]. The second, in 296.64°C, is probably related to the anticipation of the drug fusion process. The two endotherms, at 405.53 and 430.63°C, demonstrate the sample decomposition process. These differences point to an interaction between the substances in the mixture (Table 4/Fig 3D).

Five events were observed in the DTA curve clindamycin, the first, of endothermic nature, which occur at temperatures of 109.00°C, concern the dehydration of the sample. The event at 148.97°C, probably related to the delayed drug fusion process, should occur in the temperature range of 141 to 143°C [95]. From the peak observed at 170.18°C, the process of drug decomposition begins. Moreover, from the curve of the mixture, only endothermic events occurred, the first at a temperature of 101.86°C, referring to the loss of moisture in the mixture. The second, at 147.23°C was probably due to the retardation of the drug fusion process. And the third, of 204.84°C, was also attributed to the delay in the onset of decomposition in the mixture. The exothermic peaks present in the drug and the mixture were removed, indicating a possible incompatibility between the drug and the nebulized extract of *P*. *pyramidalis* (Table 4/Fig 3E).

In the DTA curve of nitrofurantoin, there was an endothermic event at 272.07°C only. The absence of previous events indicates that the sample was completely free from humidity. The first observed event is related to the early decomposition of the drug, which should take place in temperatures above 270°C [95]. There was still a last endothermic peak at 307.84°C, ending the process of decomposition. In the mixture two exothermic events were observed. The first, at 263.06°C, related to the anticipation of the decomposition process of nitrofurantoin. And the other, in 423.83°C, corresponded to the extract degradation. These events indicate that there should probably be a strong incompatibility between the drug and the substances present in the extract (Table 4/Fig 3F).

The DTA curve of ceftriaxone showed a endothermic event at 80.90°C, attributed to the dehydration process of the sample. The second, at 148.22°C, was probably responsible for initiating the melting/decomposition process of the drug, given that ceftriaxone melts with decomposition when heated to over 155°C [95,100]. The last exothermic peak, at 270.27°C, corresponds to the continuation of the drug decomposition process [101]. The DTA curve of the binary mixture ceftriaxone + extract showed two endothermic events and three exothermic. The first endotherm, of 154.40 and 85.12°C, corresponds to the first two peaks observed in the drug, as does the first exotherm, which occurred at 273.23°C, though with a delay. The second exothermic peak, at temperature 356.14°C, is probably the first peak decomposition extract that was anticipated. And, the last peak exotherm, at 546.50°C, corresponds to the lagging end of the extract decomposition process (Table 4/Fig 3G).

The gentamicin curve showed three endothermic peaks. The first of the sample, to 75.05°C, is related to water loss. The second and third peaks occurred at 252.84°C and 299.71°C. According to the Merck Index [95], the melting point of gentamicin sulfate is between 218 and 237°C. Aquino et al. [102] reported that gentamicin sulphate, the raw material, is characterized by a range of endothermic peaks related to the fusion of the different isoforms. In the curve of the binary mixture, three endothermic peaks were observed, all three of which correspond to the drug. The first peak occurred at 67.93°C, while the second and third occurred at 252.46 and 299.15°C, respectively. The temperatures of the last two peaks came very close to the peaks of the drug (Table 4/Fig 3H).

The DTA curve of Cefepime presented three endothermic peaks and an exothermic. The first two occurred at 50.13°C and 117.38°C and are related to the sample moisture loss. The third, 182.04°C in the drug, is linked to the fusion process that occurs at close to 182°C [103]. The last exothermic peak, at 645.78°C, is associated with the drug degradation process. In Cefepime + extract mixture, the first three peaks of the drug were maintained, occurring at 69.05, 108.11, and 186.27°C, respectively. With respect to the last peak, there was a delay in the melting point of the drug (Table 4/Fig 3I).

Chloramphenicol has two exothermic events at 184.87 and 279.09°C, probably linked to the melting and decomposition of the drug process. These results contrast with those found by Macedo [104], in which the fusion process of Chloramphenicol was observed in the 155.2°C exothermic event, characteristic of the drug decomposition that occurred at 244.1 and 257.8°C (Table 4/Fig 3J).

#### Thermogravimetry (TG)

**Fig 4:**
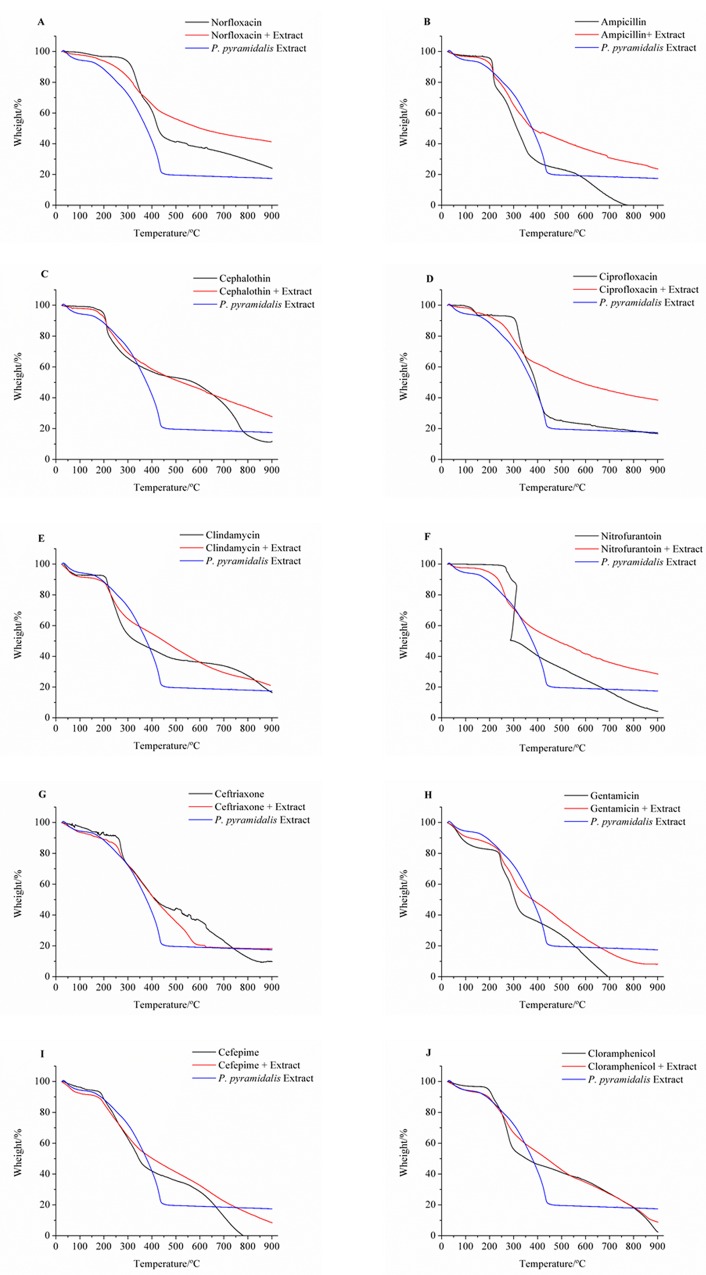
TG curves of nebulized extract of *P*. *pyramidalis*, antimicrobials and their binary mixtures. (A) Norfloxacin, (B) Ampicillin, (C) Cephalothin, (D) Ciprofloxacin (E) Clindamycin, (F) Nitrofurantoin, (G) Ceftriaxone, (H) Gentamicin, (I) Cefepime, (J) Chloramphenicol.

In the TG curves of the samples, their thermal decomposition processes were observed. The first, when it occurs at temperatures up to 100°C, refers to the loss of moisture from the sample, which, in the case of the nebulized extract of *P*. *pyramidalis*, was 6.36%. The second step is assigned to the main stage of decomposition. It indicates the beginning of the degradation process, which for this extract occurred at a temperature of 208.99°C, with a mass loss of 14.67% (Table 5/ Fig 4). During this step, many chemical chains were broken, probably caused by carbon dioxide, other gases, and novel compounds, which join to form more stable compounds. They subsequently decompose at higher temperatures. From the second stage, there is a gradual mass loss, corresponding to the whole thermal decomposition of the sample.

**Table 5:** TG Data relating to the stages of decomposition of nebulized extract, antibiotic and their binary mixtures.

Samples	Decomposition Steps	T_Peak_ (°C)	T_Onset_ (°C)	T_Endset_ (°C)	Mass loss (%)
	1st	47.22	46.50	55.58	6.36
ENPp	2nd	208.99	205.35	251.87	14.67
	3rd	372.83	370.02	429.47	58.84
	1st	49.33	56.42	72.74	7.49
	2nd	213.52	206.36	226.52	6.32
NOR	3rd	327.44	315.94	352.93	32.98
	4th	402.99	361.52	405.21	34.32
	1st	61.28	38.93	70.41	8.24
	2nd	195.49	191.55	220.03	7.28
NOR + ENPp	3rd	309.93	309.47	350.87	25.28
	4th	412.53	439.86	454.78	27.21
	5th	579.67	579.15	628.55	21.25
	1st	214.03	209.52	219.07	21.54
AMP	2nd	309.64	282.31	363.28	49.45
	3rd	630.61	612.64	688.57	25.17
AMP + ENPp	1st	210. 24	204.34	219.26	12.05
	2nd	324.42	275.73	372.71	35.87
	1st	135.48	130.16	148.40	5.27
	2nd	318.68	313.65	328.46	23.34
CIP	3rd	405.29	397.04	430.70	43.86
	1st	123.77	122.74	129.57	3.72
	2nd	264.45	278.45	299.12	25.95
CIP + ENPp	3rd	290.34	256.13	338.95	35.23
	4th	457.34	423.66	455.81	20.25
	1st	271.02	264.20	282.79	6.43
NIT	2nd	303.75	303.21	319.25	42.78
	3rd	376.93	352.38	415.19	19.13
NIT + ENPp	1st	247.51	236.47	270.91	22.50
	2nd	322.51	319.26	353.90	18.26
	1st	210.74	206.72	218.71	28.345
CFL	2nd	341.06	298.46	352.20	14.764
	3rd	719.30	697.68	791.70	39.79
	1st	207.79	201.62	219.15	27.97
CFL + ENPp	2nd	389.34	324.64	453,68	11.98
	3rd	610.88	589.22	649.52	4.96
	1st	46.69	42.78	58.64	6.98
CLI	2nd	250.42	207.55	272.20	55.29
	3rd	766.75	755.71	817.99	9.05
	1st	47.76	36.61	67.85	8.49
CLI + ENPp	2nd	229.07	227.72	259.92	28.32
	3rd	438.59	335.54	457.74	34.06
	1st	269.98	263.73	276.15	18.05
CFO	2nd	375.72	322.28	433.14	30.42
	3rd	717.90	705.93	736.42	18.14
	1st	68.76	43.99	79.59	7.22
CFO + ENPp	2nd	261.73	249.90	337.64	21.17
	3rd	408.28	398.03	466.95	53.87
	1st	59.95	44.38	78.07	11.59
	2nd	244.75	244.38	251.95	14.95
GEN	3rd	292.52	286.43	312.20	24.23
	4th	543.41	535.33	624.33	40.98
	1st	67.13	49.74	83.01	9.48
	2nd	242.30	238.64	248.33	7.22
GEN + ENPp	3rd	280.31	233.61	288.28	20.85
	4th	478.04	322.43	494.81	47.07
	1st	60.45	60.19	89.23	3.63
CPM	2nd	247.18	189.01	273.26	49.05
	3rd	667.30	626.11	755.86	39.37
	1st	56.40	46.89	75.56	6.29
CPM + ENPp	2nd	248.02	192.76	303.48	39.55
	3rd	647.30	566.36	740.84	30.48
	1st	47.86	36.27	62.29	2.74
	2nd	203.63	194.03	214.24	7.68
CLO	3rd	264.21	260.75	288.74	33.06
	4th	346.58	303.51	354.01	16.73
	5th	641.83	583.70	695.46	35.69
	1st	56.61	35.22	83.65	6.15
	2nd	199.55	198.99	215.83	6.38
CLO + ENPp	3rd	257.07	251.88	286.43	21.96
	4th	406.45	316.88	434.58	26.16
	5th	743.16	729.41	859.19	25.39

NOR = Norfloxacin; AMP = Ampicillin; CFL = Cephalothin; CIP = Ciprofloxacin; CLI = Clindamycin; NIT = Nitrofurantoin; CFO = Ceftriaxone; GEN = Gentamicin; CPM = Cefepime; CLO = Chloramphenicol.

At the end of the decomposition process, which generally occurs above 400°C, a mineral residue is present. This residue corresponds to the ash content of the sample, which in the case of *P*. *pyramidalis* extract was 58.84%. It occurred at a temperature range from 429.47 to 370.02°C. With this residue was the EEPp high, caused by the extract being dried using a 20% colloidal silicon dioxide, an amorphous silica that only degrades at temperatures above 1600°C [105].

The drugs cephalothin, ceftriaxone, ciprofloxacin, clindamycin, and cefepime showed a three-step thermal decomposition, and the degradation stages for these samples began in 206.36 (CFL), 263.84 (CFO), 130.16 (CIP), 207.55 (CLI), and 189.01°C (CPM). The antimicrobials norfloxacin, ampicillin, and nitrofurantoin had four steps. The first step starts at temperatures of 206.36 (NOR), 209.52 (AMP), 236.47 (NIT), and 244,38°C (GEN). Chloramphenicol has five stages of decomposition, the first one starting to 194.03°C (Table 5/ Fig 4).

The TG curves of the binary mixtures showed that the mixtures produced with all the drug extracts anticipated the degradation process, except for cefepime. However, when comparing the curves of gentamicin, with its respective binary mixture, we observed that the difference between the temperatures at which the sample began decomposition processes is very small.

## Conclusion

The results of this study indicate that the extract of the *P*. *pyramidalis* bark has significant antioxidant action on the radical DPPH. The extract showed no significant antimicrobial activity against multirresistant strains; however. When it was combined with certain synthetic antibiotics. Its MIC was significantly reduced. The TG/DTA curves of the antimicrobials and their binary mixtures with the extract indicate a possible physicochemical interaction between the extract and the antibiotic. Whose mixture with the extract showed no additive effect in microbiological assays. This interaction can be confirmed by additional techniques such as X-ray diffraction. and FT-IR.

The nebulized extract of *P*. *pyramidalis* has significant potential for use as an adjuvant component formulation for use in antimicrobial therapy and is a promising alternative in combating multidrug-resistant bacteria.
